# Incidence of Early and Late-Onset *Clostridioides difficile* Infection following Appendectomy Compared to Other Common Abdominal Surgical Procedures

**DOI:** 10.1155/2022/8720144

**Published:** 2022-06-07

**Authors:** K. W. Sadik, T. Hranjec, H. J. R. Bonatti, R. G. Sawyer

**Affiliations:** ^1^Department of Surgery, Div. of Reconstructive Surgery, Guthrie Clinic, Sayre, PA, USA; ^2^Department of Surgery, University of Virginia Health Systems, Charlottesville, VA, USA; ^3^Division of Transplantation, Department of Surgery, Milton S, Hershey Medical Center, Hershey, PA, USA; ^4^Meritus Surgical Specialists, Hagerstown, MD, USA; ^5^Western Michigan University School of Medicine, Kalamazoo, MI, USA

## Abstract

**Introduction:**

*Clostridioides difficile* associated diarrhea (CDAD) is a major public health issue. The appendix may function as a reservoir for the intestinal microbiome, which may repopulate the intestine following enteric infections including CDAD. *Patients/Methods*. This retrospective cohort study includes a total of 12,039 patients undergoing appendectomy, hemicolectomy, and cholecystectomy at a single center between 1992 and 2011 who were diagnosed with early and late-onset CDAD and were followed for a minimum of two years.

**Results:**

Cumulative CDAD rates were 2.3% after appendectomy, 6.4% after left and 6.8% after right hemicolectomy, and 4% after cholecystectomy with a median onset of 76 (range 1–6011) days after the procedure. Median time to CDAD onset was 76 days after appendectomy, 23 days after left, 54 days after right hemicolectomy, and 122 days after cholecystectomy (*p* < 0.05). Late-onset CDAD (>1 year) was significantly more common following appendectomy (37%) and cholecystectomy (39%) than after left (17%) and right (21%) hemicolectomy. Significant differences in age, gender, complication rate, and length of hospitalization between the four groups need to be considered when interpreting the results.

**Conclusion:**

The incidence of CDAD after various abdominal surgeries ranged between 2% and 7% in this study. Whereas, hemicolectomy patients had predominantly early onset CDAD, and appendectomy and cholecystectomy may increase the risk for late-onset CDAD. Appendectomy per se does not seem to increase the risk for late-onset CDAD.

## 1. Introduction

*Clostridioides difficile* [1] associated diarrhea (CDAD) is linked to a loss of microflora due to antibiotic treatment [2]. The appendix seems to function as a reservoir for the individual microbiome, allowing for inoculation of native flora into the colon after disruptions by enteric infections [3]. The appendix's harbors high concentrations of multicellular, surface-adherent communities of microorganisms encased in a matrix of extracellular polysaccharide biofilm [4, 5], which provides protection for cells within the appendix and allows microorganisms to withstand exposure to environmental stress such as antibiotics [6, 7]. Parts of the appendix biofilm regularly slough off and flow down the digestive tract continuously repopulating the microbiome [3].

CDAD is one of the most common nosocomial infections but may also cause enteric disease without exposure to antibiotics [8]. CDAD is an independent predictor of increased hospital length and mortality as well as costs [9]. Retrospective studies have shown a 0.41% early postoperative CDAD infection rate in the national appendectomy population [9]. Only limited data on *C*. *difficile* infection risk outside of the pressures of the early postoperative period with exposure to antibiotics are available. *C*. *difficile* has even been reported to cause acute appendicitis with immunosuppression being a contributing factor [10, 11]. Immunocompromised surgical patients have the highest risk to develop CDAD including relapsing disease [12–18]. Of note, recent exposure to metronidazole, which until recently was a first line treatment for CDAD, does not protect against CDAD [19]. As *C*. *difficile* infection manifests mainly in the colon, it would be of interest if removal of parts of the colon and removal of the appendix impacts the risk for CDAD, especially when taking immunosuppression and perioperative factors including antibiotic exposure out of the equation [20, 21]. If the appendix functions as a reservoir for our protective microbiome, removal of the organ may have a lasting impact on the risk for subsequent enteric infections, especially CDAD and severity of illness [22]. Cholecystectomy may also have an impact by changing the natural flow of bile in the digestive system [23, 24]. With the wide spread of *C*. *difficile* in the hospital environment, even relatively small abdominal surgical procedures may increase the risk for *C*. *difficile* colonization especially if antibiotics are administered [25].

The aim of this study was to compare the incidence, median time to onset, and pattern of frequency over time of CDAD in a large cohort of patients who underwent appendectomy, cholecystectomy, and left or right hemicolectomy. By extending the follow-up period, we aimed to include remote *C*. *difficile* infections that developed unrelated to obvious perioperative factors of the index procedure [9, 26].

## 2. Patients and Methods

A retrospective cohort study was performed using the University of Virginia Health System's electronic medical record (EMR) and paper chart archiving systems. After obtaining IRB approval, all patients undergoing open and laparoscopic left hemicolectomy (ICD: 45.75, 17.35), open and laparoscopic right hemicolectomy (ICD: 45.73, 17.35), open and laparoscopic appendectomy (ICD: 44950, 44955, 44960, 44970, 47.0, 47.01, 47.1, 47.11, 47.19, 49315, 56315, A44950, A44955, A44960, A49315, A56315), and open or laparoscopic cholecystectomy (ICD 9 code: 51.2, 51.21, 51.22, 51.23, 51.24, 54.21, 64.41) were retrieved from the University of Virginia Health System database between the years 1992 and 2011 allowing for a minimal 2-year follow-up time for diagnoses of postoperative CDAD. This dataset was cross-referenced for all patients who had ELISA proven *C*. *difficile* infections (ICD: 008.45) after the aforementioned operations.

The time frame for entering into the study was limited for several reasons. In 2011, testing for *C*. *difficile* was changed to a PCR-based method, which may have changed total annual counts and by this cause a selection bias. Also in 2011, a laparoscopic colorectal program was started, which may have caused another issue with cohorting. Finally, the IRB approval was limited to the original time frame.

Age, sex, time of operation, laparoscopic versus open, time to CDAD, time of resolution of CDAD (negative ELISA) [27, 28], preoperative and postoperative non-CDAD infections including urinary tract infection, pneumonia, and surgical site infection, recurrence of CDAD, length of hospital stay, and whether the patient is deceased were retrieved from the hospital archives and documented with full confidentiality maintained during retrieval and analysis of data. All timed data were censored. Recurrence was defined as any positive ELISA for CDAD occurring after resolution of initial postoperative CDAD (negative ELISA).

### 2.1. Exclusion Criteria

All patients carrying a diagnosis of CDAD 30 days prior to their operation were excluded. In addition, those who were considered immunocompromised (transplant recipients, patients on steroids, and HIV-positive individuals) at the time of operation and patients with inflammatory bowel disease were all excluded from the study as were patients with a history of total colectomy.

### 2.2. Statistical Analysis

Descriptive statistics for demographic data are reported. Continuous are presented as a mean ± standard deviation (SD) and/or median with range and compared using the analysis of variance (ANOVA) and/or nonparametric Kruskal–Wallis test. Categorical variables are displayed as percentage of the parameter and were analyzed using the chi-square or Fisher's exact test. Analyses were performed using the SPSS or SAS statistical software program (Version 9.1.3 for Windows; SAS, Institute, Cary, NC).

## 3. Results

### 3.1. Demographics (Table 1)

There were 4578 patients in the appendectomy, 357 patients in the left hemicolectomy, 1081 patients in the right hemicolectomy, and 6023 patients in the cholecystectomy group. The median ages were significantly different between the four groups with appendectomy patients being the youngest at 23 (range 0.5–89) years, whereas hemicolectomy patients were the oldest with median ages around 60 years. In the appendectomy and hemicolectomy groups, male/female ratio was approximately 1/1; whereas, in the cholecystectomy group, 66% of patients were female (*p*=0.03). In total, 59% of cholecystectomy and 55% of appendectomy cases were performed laparoscopically; the vast majority of hemicolectomies were performed open. Indications for colectomies included diverticular disease and malignancies approximately 35% each and inflammatory bowel disease 20%; the remaining 10% accounted for various less common indications such as ischemia, colonic volvulus, pseudoobstruction, trauma, and others. Hospital stay was median 8 and 10 days for right and left hemicolectomy and 2 days for appendectomy and 3 days for cholecystectomy patients (*p* < 0.05).

### 3.2. CDAD (Table 1)

Presurgical non-*C*. *difficile* infection rates including urinary tract infections, pneumonias, and wound infections ranged between 1% (appendectomy group) and 3% (right hemicolectomy group). Postsurgical non-*C*. *difficile* infection rates ranged between 6% (appendectomy group) and 25% (left hemicolectomy group).

**Figure 1:**
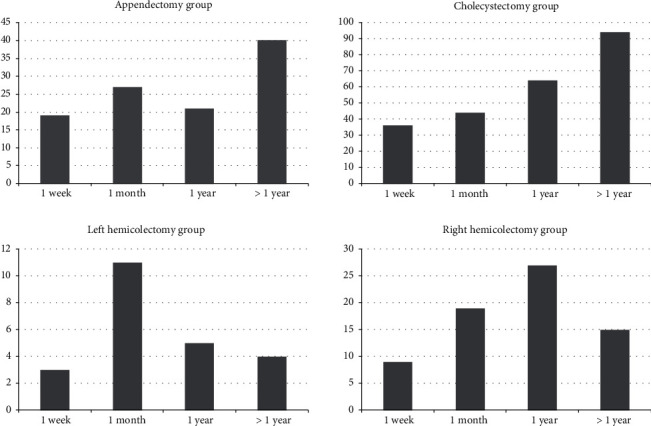
CDAD cases for the 4 groups according to the postoperative period (1^st^ week, 1^st^ month, 1^st^ year, and >1 year).

In total, 444 patients were diagnosed with postoperative CDAD. The cumulative incidence of postoperative CDAD was 6.8% in the right hemicolectomy and 6.4% in left hemicolectomy groups, 4.0% in the cholecystectomy group, and 2.3% in the appendectomy group. Median time to CDAD was 76 days in the appendectomy group and 122 days in the cholecystectomy group as compared to 23 days in the left hemicolectomy group and 54 days in the right hemicolectomy group. In the hemicolectomy groups, CDAD occurred in approximately 80% within one year postsurgery. In contrast, more than one-third of CDAD cases were diagnosed beyond one year after surgery in the appendectomy and cholecystectomy groups. In the hemicolectomy groups, a steady decline of likelihood to being diagnosed with CDAD was found; appendectomy and cholecystectomy patients had a lower risk for early CDAD but much higher probability to develop the disease after one year following surgery.  Figure 1 shows CDAD cases diagnosed during 1^st^ week, 1^st^ month, 1^st^ year, and thereafter according to the four study groups.

CDAD recurrence rates ranged between 4.3% and 5.5% for patients after appendectomy and colectomy, but it was only 1.2% after cholecystectomy (*p*=0.06).

## 4. Discussion

The incidence of early postoperative CDAD following appendectomy and cholecystectomy is lower than after left and right hemicolectomy groups. In all groups, a peak in CDAD cases was observed during the immediate postoperative period. In the hemicolectomy groups, this peak was most pronounced, and thereafter, a steady decline of CDAD cases over time was found. In contrast, after appendectomy and cholecystectomy, one-third of CDAD cases were diagnosed later than one year after surgery. However, the study was unable to demonstrate that removal of the appendix per se increases the risk for late-onset CDAD.

The overall incidence of CDAD in our cohort is comparable to previously published data [9, 25]. Currently, in the US, almost 4% of patients undergoing abdominal surgery will develop CDAD, and the vast majority will have this infectious complication during the early postoperative period. The factors leading to these concerning data are well described, and prolonged antibiotic exposure, use of proton pump inhibitors, and poor hospital hygiene are amongst the modifiable factors [9, 25]. However, changing demographics such as an increase in immunocompromised individuals and elderly surgical patients carrying significant comorbidities may be underestimated contributors. Patient selection would be a possible approach; however, denying patients at high risk for CDAD antibiotics or surgical procedures is not a viable option, and CDAD cannot be prevented by antibiotics including metronidazole or oral vancomycin [19].

With regard to lifelong risk for CDAD, again, multiple factors have been studied, but very little data are available addressing the anatomical and functional changes following abdominal surgeries [25, 26, 29–32].

The study has multiple limitations. Most importantly, finding a model to study the problem of late-onset CDAD attributable to appendectomy is very difficult. In addition, this is a single-center study using of a historical surgical cohort with a limited time frame. Loss to follow-up of patients needs to be considered; false positive and negative test results with diagnosis based on ELISA and issues with coding are additional drawbacks. During the early study years, a higher number of CDAD cases may not have been diagnosed. Finally, the emergence of a hypervirulent strain during the study period potentially changing the pattern of CDAD could not be included in our calculations [21]. However, most of these factors should have impacted all four groups in a similar fashion.

Many changes in patient care have occurred during the study period and this has affected the four groups in different fashion. One of the most impacting factors is the emergence of minimal invasive surgery, especially the development of advanced laparoscopic colorectal surgery. The vast majority of patients in this study had open hemicolectomies. Whereas, the benefits of laparoscopic approach may impact the incidence of early CDAD, it does not alter the underlying disorders that increase the risk of CDAD. This includes patients with colorectal cancer who will be exposed to chemotherapy, elderly individuals with comorbidities suffering from ischemic and adynamic colon diseases and patients with diverticulitis with often repeat courses of antibiotic therapy. Some of these conditions impact future exposure to antibiotics, which is the most important risk factor to develop CDAD. For this reason, we were surprised that in our study population, the hemicolectomy patients had a lower risk for late-onset CDAD than patients after appendectomy and cholecystectomy.

Another clinical factor that should be considered is developments in the management of acute appendicitis. CT scanning has been accepted as the new standard in diagnosis with ultrasound being a viable alternative especially in the pediatric setting. This has caused a decline in the overall number of appendectomies, and again today, the vast majority of appendectomies are done using laparoscopy—in our cohort, 45% of patients had open appendectomy. Finally, nonoperative management of appendicitis has been accepted as an alternative to surgery for many patients [33]. Antibiotic therapy is also now widely used for 1^st^ and 2^nd^ episodes of uncomplicated diverticulitis. Only limited data on the rate of CDAD in patients treated with antimicrobial agents for acute diverticulitis and appendicitis are available [34].

To conclude, the incidence of CDAD after various abdominal surgeries ranged between 2% and 7% in this study. Patients after hemicolectomy developed predominantly early onset CDAD triggered by perioperative factors, whereas appendectomy and cholecystectomy may render patients to be more susceptible to develop CDAD long-term especially if exposed to antibiotics [35]. However, in this study, we were not able to demonstrate that appendectomy per se could increase the risk for late-onset CDAD, and the role of the appendix with regard to CDAD has been questioned in a recent article suggesting that fecal transplants may be a promising strategy [36].

## Figures and Tables

**Table 1:** Demographic and clinical data according to the four groups.

	Appendectomy	Right colectomy	Left colectomy	Cholecystectomy	Total
*n*	4578	1081	357	6023	12039
% laparoscopic	55%	2%	1%	59%	51%
Median age (range)	23 (0.5–89) years	59 (0.5–89) years	62 (0.5–88) years	48 (0.5–89) years	41 (0.5–89) years
% female	53%	50%	49%	66%	59%
% Caucasian	77%	79%	77%	81%	79%
Median LOS (range)	2 (0.5–441) days	8 (0.5–1370) days	10 (1–214) days	3 (0.5–327) days	3 (0.5–1370) days
Pre-op infection	1%	3%	2%	2%	2%
Post-op infection	6%	18%	25%	9%	9%
*n,* CDAC	107	73	23	241	444
% CDAC	2.3%	6.8%	6.4%	4.0%	3.7%
Median time to CDAC (range)	76 (1–6011) days	54 (2–4621) days	23 (3–5633) days	122 (1–5383) days	76 (1–6011) days
CDAC onset after 1 year	40 (37%)	15 (21%)	4 (17%)	93 (39%)	154 (34%)

## Data Availability

The data used to support this study are not available due to HIPPA restrictions.

## Abbreviations

LOS: Length of stay
OP: Operative
CDAD: *Clostridioides difficile* associated colitis.

## References

[1] Guh A. Y., Kutty P. K. (2018). *Clostridioides difficile* infection. *Annals of Internal Medicine*.

[2] Baxter R., Ray G. T., Fireman B. H. (2008). Case-control study of antibiotic use and subsequent *Clostridium difficile*-associated diarrhea in hospitalized patients. *Infection Control and Hospital Epidemiology*.

[3] Randal Bollinger R., Barbas A. S., Bush E. L., Lin S. S., Parker W. (2007). Biofilms in the large bowel suggest an apparent function of the human vermiform appendix. *Journal of Theoretical Biology*.

[4] Donlan R. M., Costerton J. W. (2002). Biofilms: survival mechanisms of clinically relevant microorganisms. *Clinical Microbiology Reviews*.

[5] Hall-Stoodley L., Costerton J. W., Stoodley P. (2004). Bacterial biofilms: from the natural environment to infectious diseases. *Nature Reviews Microbiology*.

[6] Ceri H., Olson M. E., Stremick C., Read R. R., Morck D., Buret A. (1999). The calgary biofilm device: new technology for rapid determination of antibiotic susceptibilities of bacterial biofilms. *Journal of Clinical Microbiology*.

[7] Harrison J. J., Rabiei M., Turner R. J., Badry E. A., Sproule K. M., Ceri H. (2006). Metal resistance in candida biofilms. *FEMS Microbiology Ecology*.

[8] Deshpande A., Pasupuleti V., Thota P. (2013). Community-associated *Clostridium difficile* infection and antibiotics: a meta-analysis. *Journal of Antimicrobial Chemotherapy*.

[9] Zerey M., Paton B. L., Lincourt A. E., Gersin K. S., Kercher K. W., Heniford B. T. (2007). The burden of *Clostridium difficile* in surgical patients in the United States. *Surgical Infections*.

[10] Brown T. A., Rajappannair L., Dalton A. B., Bandi R., Myers J. P., Kefalas C. H. (2007). Acute appendicitis in the setting of *Clostridium difficile* colitis: case report and review of the literature. *Clinical Gastroenterology and Hepatology*.

[11] Pham C. D., Hua D. T. (2021). *Clostridium difficile* appendicitis in an immunocompromised patient: a case report and review of the literature. *Journal of Medical Case Reports*.

[12] Albright J. B., Bonatti H., Mendez J. (2007). Early and late onset *Clostridium difficile*-associated colitis following liver transplantation. *Transplant International*.

[13] Bonatti H. J. R., Metzger R., Swenson B. R. (2014). Solid organ recipients are at increased risk for recurrent *Clostridium difficile* colitis. *European Surgery*.

[14] Micic D., Yarur A., Gonsalves A. (2018). Risk factors for *Clostridium difficile* isolation in inflammatory bowel disease: a prospective study. *Digestive Diseases and Sciences*.

[15] Peretz A., Ben Shlomo I., Nitzan O., Bonavina L., Schaffer P. M., Schaffer M. (2016). *Clostridium difficile* infection: associations with chemotherapy, radiation therapy, and targeting therapy treatments. *Current Medicinal Chemistry*.

[16] Razik R., Rumman A., Bahreini Z., McGeer A., Nguyen G. C. (2016). Recurrence of *Clostridium difficile* infection in patients with inflammatory bowel disease: the RECIDIVISM study. *American Journal of Gastroenterology*.

[17] Stelzmueller I., Goegele H., Biebl M. (2007). *Clostridium difficile* colitis in solid organ transplantation--a single-center experience. *Digestive Diseases and Sciences*.

[18] Reddy S. S., Brandt L. J. (2013). *Clostridium difficile* infection and inflammatory bowel disease. *Journal of Clinical Gastroenterology*.

[19] Metzger R., Swenson B. R., Bonatti H. (2010). Identification of risk factors for the development of clostridium difficile-associated diarrhea following treatment of polymicrobial surgical infections. *Annals of Surgery*.

[20] Brown K. A., Daneman N., Jones M. (2017). The drivers of acute and long-term care *Clostridium difficile* infection rates: a retrospective multilevel cohort study of 251 facilities. *Clinical Infectious Diseases*.

[21] Carignan A., Allard C., Pepin J., Cossette B., Nault V., Valiquette L. (2008). Risk of *Clostridium difficile* infection after perioperative antibacterial prophylaxis before and during an outbreak of infection due to a hypervirulent strain. *Clinical Infectious Diseases*.

[22] Clanton J., Subichin M., Drolshagen K., Daley T., Firstenberg M. S. (2013). Fulminant *Clostridium difficile* infection: an association with prior appendectomy?. *World Journal of Gastrointestinal Surgery*.

[23] Fehervari Z. (2017). What is the point of the gallbladder?. *Nature Immunology*.

[24] Sorg J. A., Sonenshein A. L. (2009). Chenodeoxycholate is an inhibitor of *Clostridium difficile* spore germination. *Journal of Bacteriology*.

[25] Napolitano L. M., Edmiston C. E. (2017). *Clostridium difficile* disease: diagnosis, pathogenesis, and treatment update. *Surgery*.

[26] Sanders N. L., Bollinger R. R., Lee R., Thomas S., Parker W. (2013). Appendectomy and *Clostridium difficile* colitis: relationships revealed by clinical observations and immunology. *World Journal of Gastroenterology*.

[27] Viscidi R., Laughon B. E., Hanvanich M., Bartlett J. G., Yolken R. H. (1984). Improved enzyme immunoassays for the detection of antigens in fecal specimens. Investigation and correction of interfering factors. *Journal of Immunological Methods*.

[28] Lee S. D., Turgeon D. K., Ko C. W., Fritsche T. R., Surawicz C. M. (2003). Clinical correlation of toxin and common antigen enzyme immunoassay testing in patients with *Clostridium difficile* disease. *American Journal of Gastroenterology*.

[29] Harries R. L., Ansell J., Codd R. J., Williams G. L. (2017). A systematic review of *Clostridium difficile* infection following reversal of ileostomy. *Colorectal Disease*.

[30] Seretis C., Seretis F., Goonetilleke K. (2014). Appendicectomy and clostridium difficile infection: is there a link?. *Journal of Clinical Medicine and Research*.

[31] Yong F. A., Alvarado A. M., Wang H., Tsai J., Estes N. C. (2015). Appendectomy: a risk factor for colectomy in patients with *Clostridium difficile*. *The American Journal of Surgery*.

[32] Hussain Z. I., Todd N., Adams S., Stojkovic S. G. (2012). Prevalence of clostridium difficile in excluded colons. *The American Surgeon*.

[33] Huston J. M., Kao L. S., Chang P. K. (2017). Antibiotics vs. appendectomy for acute uncomplicated appendicitis in adults: review of the evidence and future directions. *Surgical Infections*.

[34] Feuerstadt P., Das R., Brandt L. J. (2013). Diverticular disease of the colon does not increase risk of repeat *C*. *difficile* infection. *Journal of Clinical Gastroenterology*.

[35] Girard-Madoux M. J. H., Gomez de Aguero M., Ganal-Vonarburg S. C. (2018). The immunological functions of the appendix: an example of redundancy?. *Seminars in Immunology*.

[36] Joshi T., Elderd B. D., Abbott K. C. (2018). No appendix necessary: fecal transplants and antibiotics can resolve *Clostridium difficile* infection. *Journal of Theoretical Biology*.

